# Correlation of Air Permeability to Other Breathability Parameters of Textiles

**DOI:** 10.3390/polym14010140

**Published:** 2021-12-30

**Authors:** Karel Adámek, Antonin Havelka, Zdenek Kůs, Adnan Mazari

**Affiliations:** Department of Clothing, Faculty of Textiles, Technical University of Liberec, 46117 Liberec, Czech Republic; k.adamek@volny.cz (K.A.); antonin.havelka@tul.cz (A.H.); zdenek.kus@tul.cz (Z.K.)

**Keywords:** medical textiles, air permeability, water vapour, resistance, breathability

## Abstract

In the field of textile comfort of smart textiles, the breathability of the material is very important. That includes the flow of air, water and water vapours through the textile material. All these experiments are time consuming and costly; only air permeability is much faster and economical. The research is performed to find correlation between these phenomena of breathability and to predict the permeability based on only the air permeability measurement. Furthermore, it introduces a new way of expressing the Ret (water vapour resistance) unit according to SI standards as it is connected with the air permeability of garments. The need to find a correlation between air permeability and water vapour permeability is emphasised in order to facilitate the assessment of clothing comfort. The results show that there is a strong relation between air permeability and water vapour permeability for most of the textile material.

## 1. Introduction

In recent years, textile materials used for clothing purposes have been provided with new functional properties to emphasize their improved utility properties for the wearer, that is, in particular, clothing comfort, which means not only sensory comfort but also thermophysiological clothing comfort for the wearer as specified in the definitions of comfort [1]. Thermal comfort means that such thermal proportions are achieved which make a person feel neither too cold nor too warm—a person feels comfortable [2]. Thermal comfort (sometimes also thermal neutrality) refers to the condition when the environment dissipates a person‘s heat production without significant (wet) sweating [3]. Thermal comfort is the condition of mind that expresses satisfaction with the thermal environment and is assessed by subjective evaluation [4,5,6,7].

These definitions clearly highlight the importance of air and water vapour permeability to achieve clothing comfort. The presence of sweat on the skin in the form of liquid always means considerable discomfort for the wearer. Therefore, this article deals primarily with the problem of evaluation of water vapour permeability according to various standards tested at the Department of Clothing of the Technical University of Liberec. The article describes the methods of evaluation of water vapour permeability of textile materials according to standardized techniques such as the basic SGHP (sweating guarded hotplate) method [8] and inverted cup methods using standards [9]. The conclusion discusses the possibility of expressing the Ret according to SI standards in keeping with the indication of the permeability of air flowing through fabrics.

The accuracy of measurement and the correct comparison of different measurement results are affected by a number of parameters. The measurements discussed below determine the volumetric flow rate of gas, which generally depends on its temperature and pressure. The gas permeability rate of the test sample is defined as the volumetric air and water vapour flow rate. According to the gas state equation:

p · V = r · T and p = ρ · r · T.(1)

The specific volume of gas in (m^3^/kg) and its density ρ (kg/m^3^) depend on its pressure p (Pa) and temperature T (K), with so-called gas constant r (J/(kg·K)) being the constant of proportionality. As for small deviations of the state variables, their influence on the result is negligible, but in general, these influences must be taken into account. It is true that in the laboratory measurements of porous materials the pressure gradient is small, which means the effect of pressure variation on the volumetric flow rate is negligible. Conversely, when measuring dense and poorly permeable materials, the pressure gradient is larger and the measured volumetric flow rate should be converted into a selected standard state. The ideal approach is to express the gas/vapour flow rate as a mass flow rate (kg/s), since this, unlike the volumetric flow rate (m^3^/s), does not depend on the state variables of the medium being measured. In addition, variations in the ambient state (barometric pressure, temperature, humidity) objectively affect the actual volumetric flow rate, as discussed below.

### 1.1. Air Humidity Effect

Humid air is a mixture of dry air (disregarding minor elements, it is about 79% N_2_ + 21% O_2_) and water vapour (only a few grams of dry air), and its density is therefore determined by the formula according to [3] and others as

ρ = 1/T · [B/rv + φ · pp″ · (1/rp − 1/rv)],(2)

where the following applies to the humid air used here

ρ (kg/m^3^): Calculated density
B (kPa): Barometric pressure
T (K): Temperature
φ = pp/pp″ (-): Relative air humidity (its saturation with vapour) [3 and others]
pp″ (Pa) = f(T): Vapour pressure [6]
pp (Pa) = f(T,φ): Partial vapour pressure in unsaturated air
rv (J/(kg·K)): Air gas constant = 287.1
rp (J/(kg·K)): Water vapour gas constant = 461.8

This effect should also adjust the result of permeability measurement. A reference example: the density of saturated (φ = 1) air (30 °C, 100 kPa) is larger than dry air (φ = 0) by 0.019 kg/m^3^, i.e., by 1.6%.

### 1.2. Air Flow Rate Measurement

To measure the flow of air through the test sample float-type flow meters (rota meters) calibrated for the specific state of gas being measured are used. The conversion to the measurement conditions or conversion to a standard condition is more complicated here due to the measurement principle (a float of a certain specific gravity floats in the medium being measured). The conversion details are provided in [4]; the method has been applied, for instance, when measuring the consumption of compressed air and energy of air-jet weaving machines [5].

When measuring the flow of gases, their density is negligible compared to the density of the float used (metal, plastic); the general conversion is thus simplified to:

Vc = Vm · √(ρm · rm/(ρc/rc)) = Vm · √(pm/Tm · Tc/pc),(3)

where:

p (Pa): Pressure
T (K): Absolute temperature
V (m^3^/s): Volumetric flow rate
ρ (kg/m^3^): Density
r (J/(kg·K)): Gas constant
index c: Calibration condition
index m: Measurement condition

### 1.3. Viscosity Effect

Besides being affected by velocity, the flow resistance is also affected by the viscosity of the flowing medium. Table 1 below shows that, within the usual temperature range, the dynamic viscosity of air is about twice that of water vapour, but their density values differ more significantly. The kinematic viscosity (i.e., dynamic viscosity divided by density) of vapour within the 20–50 °C range is approximately 35–37 times larger than that of air. It is possible to conclude that the values are generally equal for both media.

Since gas viscosity affects its friction when flowing, thus also affecting flow resistance, it can be presupposed that increased vapour viscosity means increased resistance in the identical fabric sample and, therefore, with other conditions being equal, lower flow/permeability compared to air.

If only a few isolated permeability measurements are to be conducted for own use, it is possible to proceed disregarding the gas condition upstream of the sample and the ambient condition downstream of the sample. However, if the results obtained in different laboratories and at different times for different materials are to be compared, the ambient conditions must be taken into account and the measured values converted to a suitable standard state. The individual parameters discussed above affect the result by only a few percent, but the possible cumulative error can come close to a quite significant 10%.

### 1.4. Breathability/Permeability Parameters

An analysis of and a discussion regarding the measurement and evaluation of the air, water vapour and water permeability rates of the test samples is provided.

Breathability/permeability parameters are measured and evaluated as the volumetric flow rate of gas (m^3^/s) through the sample area (m^2^). In physics, or rather thermodynamics [7], this quantity is called the volumetric flow rate density:

m^3^/(m^2^·s) = m/s. (4)

In addition, it is probably unnecessary to introduce another name for it in the textile industry. It is worth noting again that, according to gas state Equation (1), the volume of gas depends on its temperature and pressure. The results, therefore, need to be converted to some agreed standard state, preferably using the mass flow rate density, since mass is an objective reality, independent of the state of gas being measured:

kg/(m^2^·s). (5)

### 1.5. Air Permeability

The air permeability of fabrics is defined as the volumetric flow rate (m^3^/s) per area unit (m^2^), i.e., as the velocity of flow through a surface (m/s) according to (4). In order to compare different results obtained under different conditions, it is necessary to convert the observed data to a suitable standard value.

For unknown reasons, a sample’s air permeability value is usually given only for a single pressure difference upstream and downstream of the sample being measured. For further application, it would be more appropriate to determine the so-called flow characteristic, which means to determine the flow of air (air permeability) for several set pressure gradients

V(m^3^/s) = f(Δp(Pa)), (6)

Conversely, the so-called resistance characteristic Δp = f(V). It is then possible to determine from the result the trend of the relationship thus detected.

### 1.6. Water Vapour Permeability

#### 1.6.1. Theory

Humid air is a mixture of water vapour and gases, in simple terms, oxygen and nitrogen [3]. Because these gases have similar properties, simplified calculations give their mixture as a single component, so-called dry air.

According to Dalton’s law, each component in that mixture behaves as if it were there on its own—it has the same temperature and occupies the entire volume of the mixture, with each component having its own partial pressure and its sum being equal to the pressure of the mixture (here, for example, the barometric pressure). For example, saturated air with an atmospheric pressure of 100.00 Pa and a temperature of 40 °C contains saturated water vapour (φ = 1, pp” = 8342 Pa), air with a lower relative humidity contains less water vapour (e.g., for φ = 0.9, pp = 7508 Pa). The difference in partial pressures of water vapour (here, 834 Pa) is the driving force for the water vapour to move by diffusion from a location of higher vapour pressure to a location of lower vapour pressure. The pressure of the residue in the mixture, i.e., dry air, in this example is 91,658 and 92,492 Pa, respectively. Obviously, in standard calculations, this difference is not taken into account [3].

Diffusion is the spontaneous movement of molecules between two environments with different concentrations of molecules until these concentrations become equal. Gas (vapour) particles mix with the gas (air) that was originally there until the vapour concentrations equalize. This depends on time and temperature as determined by the formula

m = δ · S/d · t · Δp(7)

where

m (kg): Diffused vapour
δ (s): Water vapour diffusion coefficient (building materials in the order of magnitude of 10^−10^)
S (m^2^): Area of flow
t (s): Time
d (m): Thickness
Δp (Pa): Pressure gradient

After adjustment it becomes

m/(S · t) = δ/d · Δp (kg/(m^2^·s) (8)

which is identical to the (mass) permeability for vapour g/(m^2^·day)

Another quantity used is diffusion resistance

R = d/δ (m/s),(9)

which is identical to the (bulk) permeability of the sample or it is the inverted value of resistance of flow through the sample according to the SGHP method. The resistances of the individual layers are added up (serially). These two quantities are identical for physics and for textiles but have different names.

For illustration, Table 2 lists the values of these pressures for the usual temperature range [3,4,5,6,7] to determine the pressure gradient when conducting measurement.

#### 1.6.2. Water Vapour Resistance—SGHP Method

Unlike the measurement of the flow of air through a sample, this method, the so-called SGHP (sweating guarded hotplate) [8], measures the resistance of the flow of water vapour through the surface of a sample. It is one of the basic evaluation methods, developed as the model of skin with a temperature of 35 °C and 100% relative humidity upstream of the sample and the equal temperature of 35 °C downstream of the sample. This isothermal measurement prevents condensation of passing vapour in the sample material in the case when the temperature downstream of the sample should be below the dew point. In practice, however, ambient conditions such as those downstream of the sample here occur perhaps in humid subtropical zones, not in temperate climates.

This method makes it possible to measure the resistance of water vapour flow, inaccurately referred to as “evaporative resistance” Ret [m^2^·Pa/W] in accordance with ISO 11092 [8]. The skin model is thermally insulated so that the heat required to make distilled water evaporate passes only towards the material being tested. The entire instrument must be housed in an air-conditioned chamber to achieve steady-state thermal and humidity conditions. The velocity of flow over the sample is constant, but it must be noted that the rate of removal of the passing vapour is largely dependent on the velocity of the ambient air. This method is definitely one of the basic standard methods for determining the thermal and evaporative resistance of fabrics.

The instrument measures the resistance of water vapour permeability—the ‘evaporative resistance’ Ret. Ret is determined as the difference in partial pressures of water vapours between the surfaces below and above the sample being measured (in the vicinity of the instrument) divided by the heat flow rate required for steady-state evaporation.

(10) $$\mathrm{Ret}=\frac{\left(p^{\prime \prime }-\mathrm{pa}\right)\;\cdot \;S}{H-∆\mathrm{He}}-\mathrm{Ret}0\;$$

where

p″ (Pa): Partial (saturated) water vapour pressure upstream of the test sample
pa (Pa): Partial water vapour pressure downstream of the test sample
S(m^2^): Measurement surface
H (W/m^2^): Heat flow rate
ΔHe (W/m^2^): Heat flow adjustment due to loss
Ret (Pa·m^2^/W): Water vapour resistance of the sample
Ret0 (Pa·m^2^/W): Adjustment − resistance of flow through cellophane = instrument constant

Ret ≤ 10 is reported to mean good water vapour permeability, with water vapour permeability being low above this value [1,9].

The water supply of constant temperature (35 °C) is separated from the sample by cellophane, which does not allow liquid water to pass through but lets through (saturated) water vapour. The cellophane provides resistance to the flow of vapour, which is considered a constant under the given measurement conditions. Thus, the resulting resistance of the two resistances in a series (cellophane + sample) is measured; after subtracting the resistance of the cellophane (this is the constant of the instrument), what remains is the unknown resistance of the sample for the vapour pressure gradient (p″ - pa) set during measurement. Thus, from one side of the sample, there is only saturated vapour, continuously produced during measurement by heating the water supply using measured power consumption.

The measured parameter represents the water vapour resistance of the sample. It is defined as the steady-state heating capacity required to achieve a steady-state flow of vapour through a fabric‘s surface at a given pressure gradient. Once SI base units have been substituted into Ret value (10) and cancelled, the result is simple—it is the inverse value of flow velocity

m^2^·Pa/W = m^2^·N/m^2/^(N·m/s) = s/m (11)

This is logical because the air permeability (breathability) of the layer being observed of the medium and the flow resistance of the same layer are inverse values. If the same/inverted values were shown in identical units, it would be possible to compare the permeability of the sample in question for vapour and for air.

Again, as is the case with air above, the value of the permeability of fabrics for water vapour is specified in theoretical papers and in business only for a single pressure gradient on the sample being measured. It would be more appropriate to determine the overall character of such water vapour permeability, the so-called flow characteristic, i.e., the water vapour flow rate for several set pressure gradients

V(m^3^/s) = f(Δp (Pa)). (12)

In practice, this means setting a certain temperature of saturated vapour upstream of the sample and several states of air of varied humidity of identical temperature downstream of the sample, for example as indicated in Table 2.

Notes:

(1) Setting the partial pressure of saturated air vapour downstream of the sample (φ = 1) is pointless since the driving pressure gradient Δp is zero in that case.
(2) Setting the partial pressure downstream of the sample to zero, i.e., absolutely dry air (φ = 0), is technically challenging; in practice, drying the air to the dew point of +3 °C—i.e., pp = 758 Pa, is commonly used. Thus, the maximum pressure gradient for a temperature of +35 °C is in fact 4866 Pa.

## 2. Hypothesis

If permeability for air and vapour is expressed by identical units, it would be possible to hypothesize whether any general correlation exists between the two media and then use it in practice. In fact, measuring the air permeability of a sample is simpler and faster than vapour permeability. To verify this, it is not enough to determine breathability at a single point—as is generally done today—is not sufficient for verification; a series of permeability measurements for both air and vapour must be carried out for an identical sample, and for a range of pressure gradients, and subsequently, a so-called flow characteristic must be plotted according to [10,11,12] and describes the trend of relationships (6). To confirm the hypothesis more reliably, measurements need to be conducted for more samples. From more such results, one could determine whether there is any statistically significant correlation between the permeability rates of the two media in the same sample.

However, it should be noted that air permeability is actually the flow of air through a sample due to the set pressure gradient, while water vapour permeability is due to diffusion.

## 3. Measurements

Currently, no comprehensive results of air and water vapour permeability measurements are available; only isolated and unrelated results have been obtained from randomly selected papers [12,13] and used to gain insight into the matter.

For the first approximation, some previously measured values were used, without verification taken from [10]. The values of resistance against vapour permeability (a firefighting suit) apply to a single, but undefined, measurement condition

R_et_ = 35–36 m^2^·Pa/W = 35–36 s/m or to a multilayer sample

R_et_ = 35–45 m^2^·Pa/W = 35–45 s/m.

The inverted values, i.e., the water vapour permeability of the sample, are, for Ret = 35, for instance, approximately

0.028 m/s = 0.028 m^3^/(m^2^·s) and, when converted to mass units is (for a saturated vapour density of ρ = 30.35 g/m^3^ at 30 °C)

0.85 g/(m^2^·s) = 3.059 kg/(m^2^·h).

For a silver-coated sample, the resistance quoted is approximately 10 times lower, that is, the flow rate is tenfold,

Ret = 4.5 m^2^·Pa/W = 4.5 s/m.

Such a result seems unlikely since an additional layer applied to the sample structure should act the other way round, increasing the resistance or decreasing the flow rate.

In the same way, the air permeability of the identical sample coated a 1–3 μm of silver was measured, resulting in 3.82 l/(m^2^·s), i.e., 0.38 m/s, which is about 13.5 times more than in the case of vapour. Vapour viscosity is 7–35 times greater than that of air; subsequently, a flow rate lower by this ratio can be expected in the same sample and under the same ambient conditions. The result for vapour therefore corresponds to the result for air to a large extent.

However, it is challenging to establish any correlation with some degree of responsibility from merely two isolated measurements, particularly when conducted and under unknown ambient conditions. It would be useful to make a series of measurements for different pressure gradients and compare the flow characteristics.

Further previously measured values of air and vapour permeability of the same samples were taken from a dissertation [11], where the measurements observe the effect of different surface treatment types (lamination). The permeability rates for air and steam are proportionally increasing, with correlation coefficients R of the regression lines used being sufficiently high.

The slope of the regression lines varies within one order of magnitude (0.0098–0.0064–0.0266) for each group. It can be assumed that, besides the physical properties of the media (specific gravity, weave, size of molecules, etc.), different types of lamination and other influences (density, weight, weave, type of yarn in woven or knitted fabrics—i.e., pore size, type of surface treatment, etc.) seem to be acting.

The absolute element of these regression lines should be zero, i.e., the mutual relationship should pass through the origin of the coordinates. There is no reason for a sample, absolutely impermeable to one medium, to be permeable to the other medium. The small values of the absolute elements shown here (0.0004–0.00005–0.0008) are probably due to some small error in measurement or evaluation and can be neglected.

**Figure 1:**
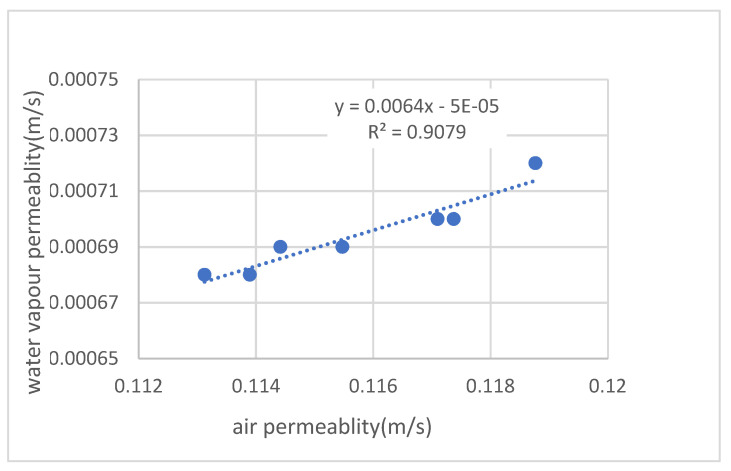
Air permeability and water vapour permeability relation.

In numerical flow simulation models [12], permeable samples of fabric are defined as the so-called “porous jump”. It is the total resistance of a sample inserted in the flow region being modelled. Thus, the method does not address the details of the flow inside the sample, i.e., the influence of individual yarns and fibrils, etc. One example of modelling such a detailed flow is the flow of resin during the filling of a mould filled with reinforcing fibres in the manufacture of composite components [14]. The air permeability and water vpour permeability relationship is shown in Figure 1.

### 3.1. Definition of Air Permeability for Various Pressure Gradients

The air permeability parameters of fabrics were determined in [12] from the result of an experimental, subsequently to be applied in a commercial program [15] for numerical breathability simulation. The necessary breathability parameters thus determined have been used in a number of investigated cases [16,17,18,19,20,21] and others.

The air permeability of the layer being monitored is generally determined by its compressive resistance against the air flow rate. Textbooks on hydromechanics state that such flow resistance consists of a linear element, typically, for example, in low-velocity seepage in soil and sponge-like structures, etc., (Darcy’s law) and a quadratic element, typically for flow through channels and the bypass flow of objects, etc. (Weissbach’s or Moody’s law). In real-life layers of interest, there is usually some kind linear combination of these two limiting cases; therefore, the commercial program assumes flow resistance Δp (Pa) as a quadratic function of flow velocity w (m/s)

Δp = A · w2 + B · w + C(13)

To proceed further, it is therefore necessary to determine the relationship between the volumetric flow rate through the sample and the pressure gradient; see also (6) above V = f(Δp), i.e., the so-called flow characteristic. For known sample flow cross-section S, the characteristic is modified to

w = V/S = f(Δp).(14)

A relationship between the pressure gradient (flow resistance) and the flow rate inverse to this function is then the developer,

Δp = f(w), (15)

i.e., the so-called resistance characteristic, already formally identical to the permeability equation. The measured points of such a resistance characteristic are then plotted on the quadratic function or the combination of the linear and quadratic elements (11).

(1) As this is a quadratic function, to capture its character it is necessary to measure at least 3 points. As a matter of course, the more points are measured, the more accurate the quadratic regression.
(2) The absolute element C in the quadratic regression should be zero since at zero velocity (w) there is zero resistance (Δp)—this is also another point of the quadratic relationship being searched for. A nonzero value of C indicates some error in the measurement or evaluation and should be detected and corrected. If, for example, the value of C is nonzero, but it is small compared to the other coefficients A, B, it can be neglected as a result.
(3) In another substitution of the relationship between flow rate and pressure gradient, the power function Δp = f(w · n) was used in [22], where n = 1.45—i.e., 1 < n < 2. It can be concluded that such a pressure resistance function also contains a combination of the first and second powers of the velocity.
(4) For geometrically simple shapes (for example, perforated sheet metal), this relationship can be obtained by numerical simulation; for complicated structures (fabrics, knitted fabrics, filter layer, etc.), it must be determined experimentally. However, specialized programs are already available for the direct simulation of the flow through even such complicated real-life layers. A wealth of useful information regarding this can be found for example in [23].

### 3.2. Measurement Results

For vapour, measurement is isothermal to prevent the condensation of vapour in the sample during measurement. Thus, it is an idealised measurement that eliminates the effect of condensation in the sample mass, which almost always occurs under real-life conditions as shown in Table 3.

**Figure 2:**
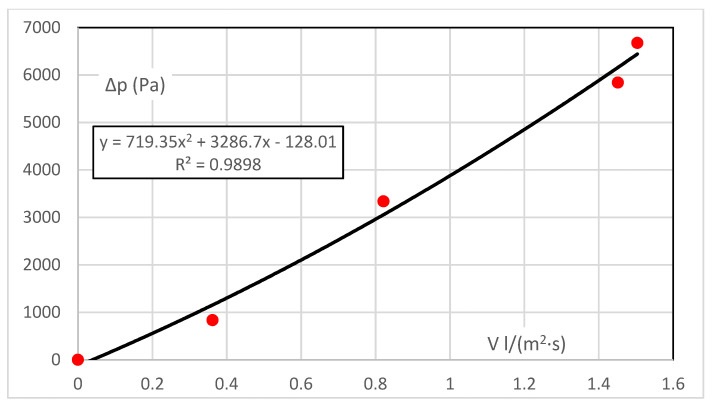
Resistance characteristic—vapour.

The range of driving pressure gradients here is up to 6.6 kPa. This is the difference in the partial pressures of the vapour upstream of the sample (constant) and downstream of the sample (varies with temperature and humidity). The resistance characteristic Δp = f(V), is shown in Figure 2. The flow characteristic, i.e., the permeability, is the inverse function of V = f(Δp).

The characteristic obtained from five measured points is almost linear, and the coefficient for the quadratic element is small. This would correspond to a diffusive flow at a very low velocity. Although the absolute element should be zero (there is zero flow resistance at zero flow), it is insignificant—the error margin of 1.7% of the measurement range can be neglected.

For air, such as in the case of vapour, the range of pressure gradients is defined by the measuring instrument available and the permeability of the same sample and is 100 Pa, i.e., an order of magnitude lower than for vapour. As for vapour, the resistance characteristic Δp = f(V), the flow characteristic, i.e., permeability, is the inverse function of V = f(Δp).

The characteristic obtained from six measured points is almost linear, and the coefficient for the quadratic term is small. Although the absolute element should be zero (there is zero flow resistance at zero flow), it is insignificant—the error margin of 0.4% of the measurement range can be neglected.

**Figure 3:**
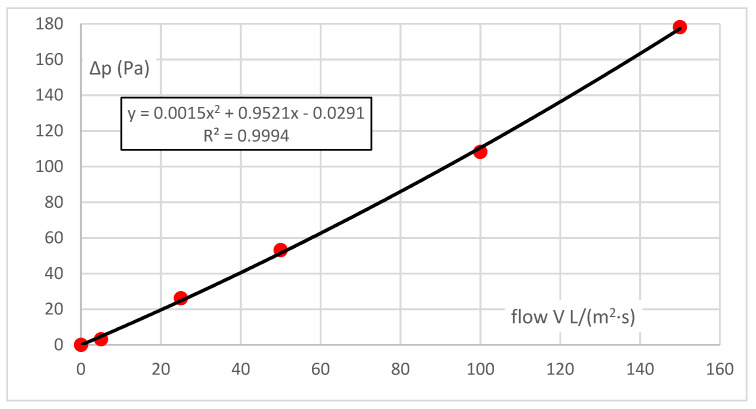
Water vapour flow at lower pressure difference.

In order to compare the permeability rates of vapour and air, the values related to vapour must be extrapolated using the established correlation function in Figure 3 into small pressures in the same way as for air (i.e., here up to about 150 Pa), and the values for air must be extrapolated using the established correlation function in Figure 2 into large pressures in the same way as for vapour (here, up to about 7 kPa). The correlations between the permeability rates of vapour and air in the same sample can subsequently be determined.

**Figure 4:**
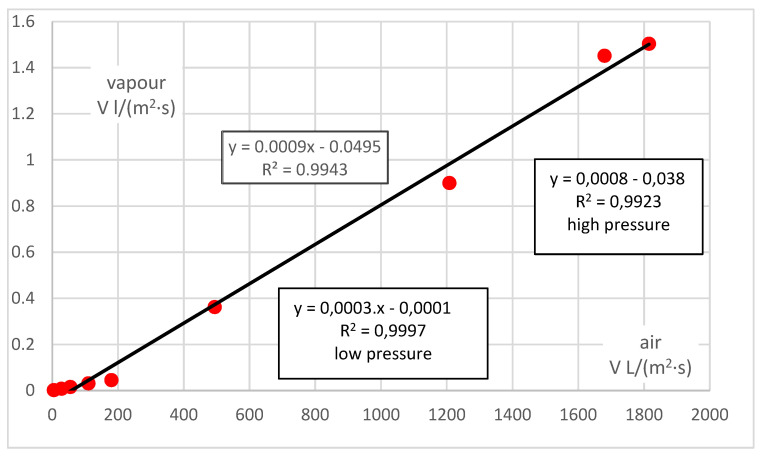
Correlation between vapour and air permeability rates—summary.

The summary result is shown in Figure 4—the correlation is linear, and the value of vapour permeability is generally negligible compared to the permeability of air, about 0.0003:1 for small pressures and about 0.0008:1 for large pressures. Taking all values together, the vapour/air permeability ratio is about 0.0009:1. In general, the absolute element should be zero (zero pressure results in zero flow for any medium). Although its value is small, the results can be affected in for low pressure values (and flows or permeability rates).

It should be noted again that the measurement values (index m) must be converted using gas state Equation (1) to some suitably chosen standard mode (index o)

Pm · Vm/Tm = po · Vo/To(16)

The result is provided only as an example; to reliably verify the correlation between air and vapour permeability, more experiments and subsequent correlations for more sample types would be required.

For isothermal models, the simple relationship for permeability V = f(p) applies, while for nonisothermal models, which better correspond to reality, the more complex relationship V = f(p,t) and V = f(p, Δx) are used, where Δx = f(t,φ) is the precipitated condensate, which depends on the temperatures and humidities during measurement.

## 4. Summary

This paper deals with the complex issue of air, vapour and water permeability, with the aim of unifying existing methods for evaluating the transport properties of fabrics as an important factor affecting clothing comfort. From the topics presented, the following can be selected:

(a) For correct physical measurements of volumetric flow, all parameters that may affect the measurement results should be reported. In particular, barometric pressure, temperature and relative humidity in the laboratory should be precisely defined.
(b) Use the correct naming convention for air permeability measurements, volumetric flow rate density (m/s) or mass flow rate density (kg/(m^2^·s)), see (6) and (7). To assess water vapour resistance (evaporative resistance), Ret simpler expression (11): m^2^·Pa/W = m^2^·N/m^2^/(N·m/s) = s/m should be used, which is the inverse value of the volumetric flow rate density m/s. The result of gravimetric measurement (12) in g/(m^2^·24 h) can easily be converted to the mass flow rate density kg/(m^2^·s) and, if necessary, subsequently to mass flow rate density (11).
(c) For the evaluation of water permeability measurements, choose the method that best simulates the actual rain resistance. Thus, the measured parameters for determining the resistance of a sample against defined rain should be the period of time until the first drop appears on the reverse of the sample and the amount of water soaked into the sample during the test. Measuring pressure in metres is nonsense; the correct statement should be “hydrostatic pressure of a column of water of height”.
(d) The determination of permeability should be based on the measured flow characteristics of permeability m (kg/s) = f (Δp (Pa)) for both air and water vapour, which provide a better indication of the nature of permeability than single-point measurements. From such more comprehensive data, an attempt is made to verify or refute the hypothesis of a correlation of flow characteristics for air and water vapour. The procedure is given for a single test sample as an example.

The individual properties of textile samples discussed above are examined in a laboratory in separation; however, in real life, the three layers of so-called functional garments, discussed in more detail in [24], interact. For example, the top layer of a so-called functional garment has to fulfil three contradictory requirements simultaneously:

- low air permeability from the outside (wind),
- high water vapour permeability from the inside (perspiration) and
- low water permeability from the outside (rain).

The various standards used in laboratory measurements assume a very high outside ambient temperature. However, in reality, during the cold season, the temperature of the very humid indoor air drops below the dew point on the reverse of the outer layer of the garment. Thus, according to the laws of nature, some of the water vapour must condense into water, but it remains inside the garment because the outer layer is impermeable to water. The garment becomes damp and the wearing sensation is not comfortable. In practice, this results in the lower thermal insulation layer of the garment becoming wet, thus reducing its thermal insulation resistance. Given the generally low thermal conductivity values of insulation, the effect of such wetting is not significant in absolute terms [24,25]. Alternatively, the condensed moisture on the reverse side of the outer layer (windbreaker) is masked by various nets.

According to the result of laboratory experiments, vapour should pass through the garment to the outside (the garment is significantly permeable to water vapour), but dry air should not (the garment is significantly impermeable to wind). Therefore, it is clear that the lab experiments are detached from reality—air (wind) does not penetrate the garment from the outside in, but vapour does penetrate the garment from the inside out. In reality, humid air is a mixture of dry air and water vapour, which partially condenses at temperatures below the dew point and does not penetrate clothing which is impermeable to water.

All methods used to determine the air, water vapour and water permeability of a sample are only laboratory methods which determine individual insulating properties. The isolated laboratory values are fantastic, as the Vendors never fail to mention, but they are far from being of practical use.

However, it must be noted that moisture dissipation through clothing is of great importance to the feeling of good clothing comfort, and any method of assessing water vapour permeability through fabrics that approximates an actual wearing experience is useful.

## Figures and Tables

**Table 1:** Some parameters of air and water vapour.

	Air [3]		Water Vapour [6]		
Temperature	10^6^·Dyn·Visc.	Density	10^6^·Dyn·Visc.	Density	Vapour Pressure
°C	kg/(ms)	kg/m^3^	kg/(ms)	kg/m^3^	Pa
0	17.168	1.252	9.216	0.0048	619
10	17.756	1.206	9.461	0.0094	1244
20	18.404	1.164	9.727	0.0173	2370
30	18.816	1.128	10.01	0.0304	4303
40	19.228	1.092	10.31	0.0512	7482
50	19.669	1.058	10.62	0.0831	12,515
60	20.111	1025			

**Table 2:** Partial vapour pressures pp (Pa) for various temperature values t (°C).

	φ (−)	1.0	0.8	0.6	0.4	0.2	0.0
t (°C)							
10		1228	982	736	491	245	0
15		1708	1366	1025	683	342	0
20		2337	1870	1402	935	467	0
25		3168	2534	1901	1267	634	0
30		4238	3390	2543	1695	848	0
35		5624	4499	3374	2250	1125	0
40		8342	6672	5004	3336	1668	0

**Table 3:** Defined and measured parameters of a sample’s vapour permeability (40 °C).

φ	%	20	30	60	90	100
pp	Pa	1668	2503	5005	7508	8342
Δp	Pa	6674	5839	3337	834	0
m	g/(m^2^·d)	6646	6415	3630	1599	0

## Data Availability

The data presented in this study are available upon request from the corresponding author.
